# Efficacy and Safety of Switching Prostaglandin Analog Monotherapy to Tafluprost/Timolol Fixed-Combination Therapy

**DOI:** 10.1155/2018/8456764

**Published:** 2018-02-21

**Authors:** Kazuyoshi Kitamura, Tatsuya Chiba, Fumihiko Mabuchi, Kiyotaka Ishijima, Shu Omoto, Fumiko Kashiwagi, Takashi Godo, Satoshi Kogure, Teruhiko Goto, Takashi Shibuya, Jhoji Tanabe, Shigeo Tsukahara, Tadaharu Tsuchiya, Toyoaki Tsumura, Takaharu Tokunaga, Osamu Hosaka, Tetsunori Saito, Kenji Kashiwagi

**Affiliations:** ^1^Department of Ophthalmology, University of Yamanashi, Chuo, Yamanashi, Japan; ^2^Department of Ophthalmology, Irumagawa Hospital, Sayama, Saitama, Japan; ^3^Department of Ophthalmology, Musashimurayama Hospital, Musashimurayama, Tokyo, Japan; ^4^Kashiwagi Eye Clinic, Kofu, Yamanashi, Japan; ^5^Godo Eye Clinic, Iida, Nagano, Japan; ^6^Kogure Eye Clinic, Showa, Yamanashi, Japan; ^7^Goto Eye Clinic, Kai, Yamanashi, Japan; ^8^Shibuya Eye Clinic, Fujikawa, Yamanashi, Japan; ^9^Tanabe Eye Clinic, Kai, Yamanashi, Japan; ^10^Tamaho Eye Clinic, Chuo, Yamanashi, Japan; ^11^Department of Ophthalmology, Fussa Hospital, Fussa, Tokyo, Japan; ^12^Mel Eye Clinic, Nishitokyo, Tokyo, Japan; ^13^Hosaka Eye Clinic, Chuo, Yamanashi, Japan; ^14^Saito Eye Clinic, Koshu, Yamanashi, Japan

## Abstract

**Purpose:**

To assess the efficacy and safety of switching from prostaglandin analog (PGA) monotherapy to tafluprost/timolol fixed-combination (Taf/Tim) therapy.

**Subjects and Methods:**

Patients with primary open-angle glaucoma, normal-tension glaucoma, or ocular hypertension who had received PGA monotherapy for at least 3 months were enrolled. Patients were examined at 1, 2, and 3 months after changing therapies. Subsequently, the patients were returned to PGA monotherapy. The examined parameters included intraocular pressure (IOP) and adverse events. A questionnaire survey was conducted after the switch to Taf/Tim therapy.

**Results:**

Forty patients with a mean age of 66.5 ± 10.3 years were enrolled; 39 of these patients completed the study protocol. Switching to Taf/Tim significantly reduced the IOP from 18.2 ± 2.6 mmHg at baseline to 14.8 ± 2.5 mmHg at 1 month, 15.2 ± 2.8 mmHg at 2 months, and 14.9 ± 2.5 mmHg at 3 months (*P* < 0.001). Switching back to the original PGA monotherapy returned the IOP values to baseline levels. Taf/Tim reduced the pulse rate insignificantly. No significant differences were observed in blood pressure, conjunctival hyperemia, or corneal adverse events. A questionnaire showed that the introduction of Taf/Tim did not significantly influence symptoms.

**Conclusions:**

Compared with PGA monotherapy, Taf/Tim fixed-combination therapy significantly reduced IOP without severe adverse events.

## 1. Introduction

Reducing intraocular pressure (IOP) using ophthalmic solutions is the primary treatment for glaucoma. However, because patients with glaucoma have relatively mild subjective symptoms until their condition reaches an advanced stage and because patients rarely notice the efficacy of ophthalmic solutions, both the adherence to and continuity of treatment are reportedly poor [1, 2]. The numbers of glaucoma patients who simultaneously use two or more IOP-lowering ophthalmic solutions are increasing in both Japan [3] and other countries [4]. Previous studies have reported that the use of fewer ophthalmic solutions decreases the burden on patients, including reducing the number of adverse effects, and improves adherence to treatment [5, 6]. Fixed-combination ophthalmic solutions have been gaining approval for clinical use, and six of these solutions are currently available in Japan. The tafluprost/timolol fixed-combination solution (Taf/Tim) has been shown to significantly reduce IOP in patients with glaucoma [7]. The amount of benzalkonium chloride (BAC) was positively correlated with ocular surface injury. Latanoprost solution contains 0.02% BAC, while travoprost solution does not contain BAC, which may result in a difference in ocular surface toxicity. Therefore, switching from monotherapy to fixed therapy may be beneficial for IOP reduction and ocular surface. However, limited information is currently available regarding the efficacy and adverse side effects of Taf/Tim among patients, including Japanese patients, with glaucoma. The objective of this study was to prospectively examine the efficacy, safety, and patient comfort of switching Japanese patients with glaucoma from latanoprost or travoprost treatment to Taf/Tim therapy.

## 2. Subjects and Methods

This study was a prospective, open-label, multicenter trial approved by the Ethics Committee of the University of Yamanashi and conducted in accordance with the Helsinki Declaration and the Ethical Guidelines for Medical and Health Research Involving Human Subjects of the Japanese Ministry of Health, Labor and Welfare. All participants provided written informed consent. Fifteen institutes participated in the trial.

### 2.1. Patients

The subjects were patients with primary open-angle glaucoma (POAG), normal-tension glaucoma (NTG), and ocular hypertension (OH) who had received prostaglandin analog (PGA) monotherapy including latanoprost and travoprost for at least 3 months and required further IOP reduction as determined by the attending physician. Patients with the following conditions were excluded from the study: chronic or recurrent uveitis; scleritis; herpes keratitis; ocular injury within 3 months prior to the study; a history of intraocular surgery within 3 months prior to the study; laser treatment within 1 month prior to the study; conditions preventing IOP measurement by applanation tonometry; and arrhythmia, asthma, or other contraindications to beta blockers. Additionally, physicians excluded any patient whose participation was regarded as inappropriate. If both eyes satisfied the enrollment and exclusion criteria, the eye with a higher baseline IOP was chosen for the study.

### 2.2. Study Protocol

**Figure 1:**
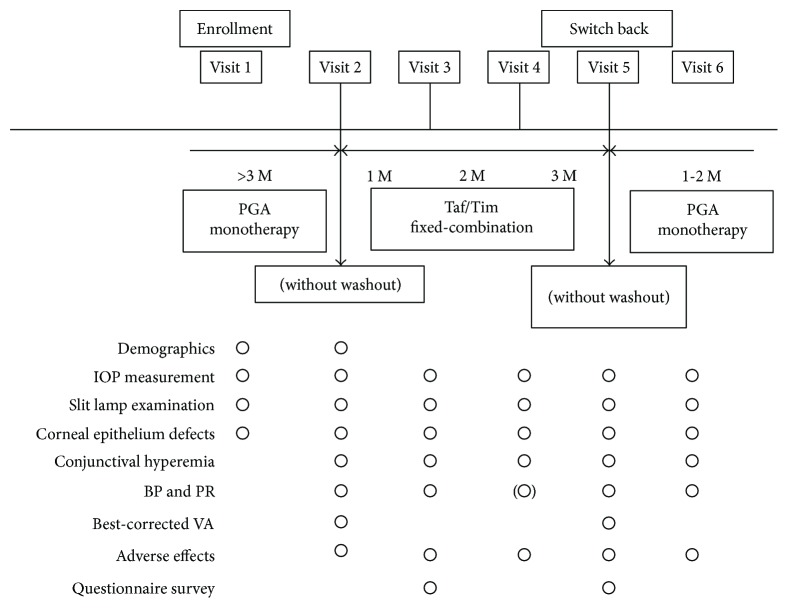
Study protocol. M: months; Taf/Tim: tafluprost/timolol; PGA: prostaglandin analog; IOP: intraocular pressure; BP: blood pressure; PR: pulse rate; VA: visual acuity.

We conducted this investigation based on a protocol established by previous studies [8, 9]; a summary of the study protocol is depicted in Figure 1. A routine ophthalmic examination was performed at all visits, and the mean IOP measured at all visits and prior to the switch to Taf/Tim therapy was defined as the baseline IOP. Taf/Tim therapy was substituted for PGA monotherapy without a washout period, and patients were examined at 1, 2, and 3 months after changing therapies. Taf/Tim was applied once every morning. IOP values show fluctuation for physiological and/or pathological reasons. It is possible that the IOP value when the ophthalmologist decides to change a glaucoma patient's medication may not always reflect an appropriate value. To eliminate this possibility, Taf/Tim was switched back to the originally administered PGA monotherapy without a washout period, and the examination was reperformed within 1-2 months. IOP measurements were performed using Goldman applanation tonometry, and the measurements were repeated twice and then averaged at all visits. IOP measurements were taken at the same time as baseline measurements. Superficial punctate keratopathy was evaluated based on a method described in a previous study [10]. Briefly, two parameters of superficial punctate keratopathy, area and density, were graded on a scale ranging from A0 to A3 and from D0 to D3, respectively. Bulbar conjunctival hyperemia was classified into four stages from grade 0 to grade 3 (0 = none, 1 = mild, 2 = moderate, and 3 = severe). Sample photo images were used to classify superficial punctate keratopathy and bulbar conjunctival hyperemia. Blood pressure and heart rate measurements were performed with the patient in a seated position at baseline; at 1, 2, and 3 months after the switch to Taf/Tim therapy; and after the switch back from Taf/Tim therapy. A questionnaire survey (Supplemental Figures 1-2) on the use and overall comfort of the ophthalmic solutions was conducted at 1 and 3 months after the switch to Taf/Tim therapy.

### 2.3. Statistical Analysis

Data were analyzed using the JMP 12.0 software program (SAS Institute, Cary, NC), and the results are presented as the means ± standard deviation (SD). Differences in the results were assessed using repeated measures analysis of variance (ANOVA), Student's *t*-test, and contingency table analysis. Correlations between two parameters were investigated using Pearson's correlation coefficient. *P* values < 0.05 were considered significant.

## 3. Results

### 3.1. Enrolled Patients

A total of 40 patients (16 men, 24 women; age: 48 to 86 years; mean age 66.5 ± 10.3 years) were enrolled in this study. One female patient was dropped due to chest pressure. Her symptom was mild, and it disappeared after she quit the use of Taf/Tim. Although a systemic examination including chest XP and electrocardiogram did not show any apparent abnormality and she had experimented with using timolol ophthalmic solution with no symptoms, we could not eliminate the possibility that her symptom was related to Taf/Tim therapy. Therefore, 39 patients completed the study protocol and were included in the analysis. Detailed characteristics are shown in Table 1.

### 3.2. Changes in IOP

**Figure 2:**
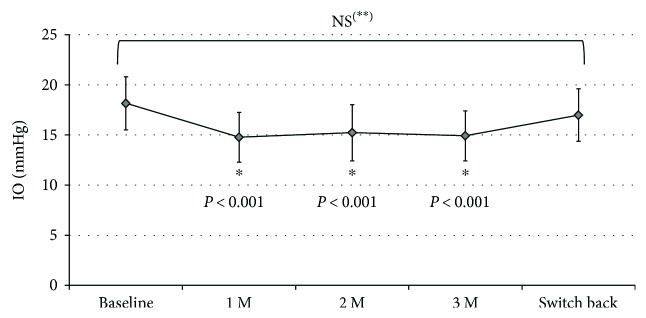
Changes in IOP during the study period. ∗ versus baseline or switch back, repeated measures ANOVA; ∗∗ baseline versus switch back, Student's *t*-test; bars: SD; M: months; IOP: intraocular pressure.

**Figure 3:**
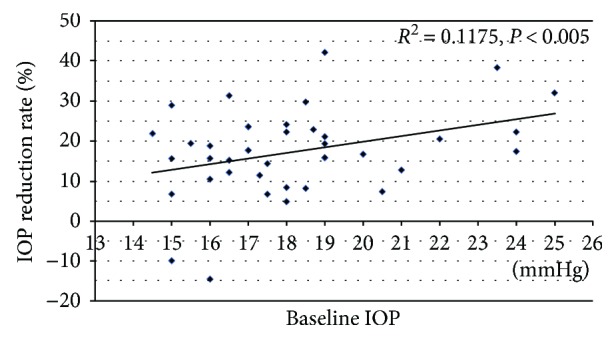
Relationship between baseline IOP and Taf/Tim-induced IOP reduction rate. IOP: intraocular pressure.

Figure 2 shows the changes in IOP throughout the study period. The baseline IOP was 18.2 ± 2.6 mmHg. The IOP values at 1, 2, and 3 months after the switch to Taf/Tim therapy were 14.8 ± 2.5, 15.2 ± 2.8, and 14.9 ± 2.5 mmHg, respectively, which were significantly reduced compared to baseline (*P* < 0.001, repeated measures ANOVA). After the switch back to PGA monotherapy, the IOP was 17.0 ± 2.6 mmHg, which was significantly higher than that measured during the Taf/Tim treatment period (*P* < 0.001, repeated measures ANOVA). The IOP after switching back to PGA was not significantly different from the baseline IOP (*P* = 0.28, Student's *t*-test). The mean IOP reduction rate during the 3-month period following the switch to Taf/Tim therapy was 17.6 ± 10.8%. As shown in Figure 3, a significant positive correlation was found between the baseline IOP and the IOP reduction.

### 3.3. Changes in Blood Pressure and Heart Rate

**Figure 4:**
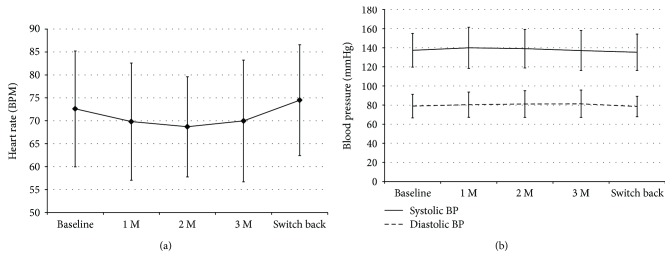
Changes in systemic circulatory parameters during the study period. (a) Heart rate. (b) Systolic and diastolic blood pressure. M: months; BPM: beats per minute; BP: blood pressure.

Compared to PGA monotherapy, Taf/Tim administration reduced the heart rate by a mean of 4.3 ± 0.96%, but this change was not significant (Figure 4(a)). No significant changes were observed in either systolic or diastolic blood pressure during the study (Figure 4(b)).

### 3.4. Topical Adverse Effects

**Figure 5:**
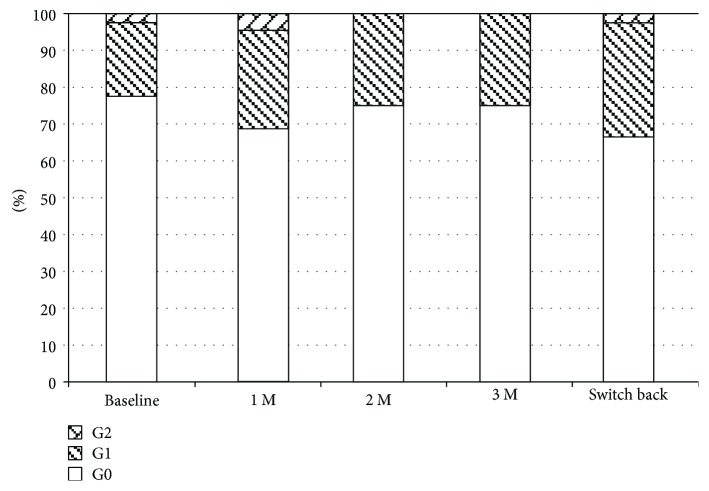
Changes in conjunctival hyperemia. M: months; G: grade.

**Figure 6:**
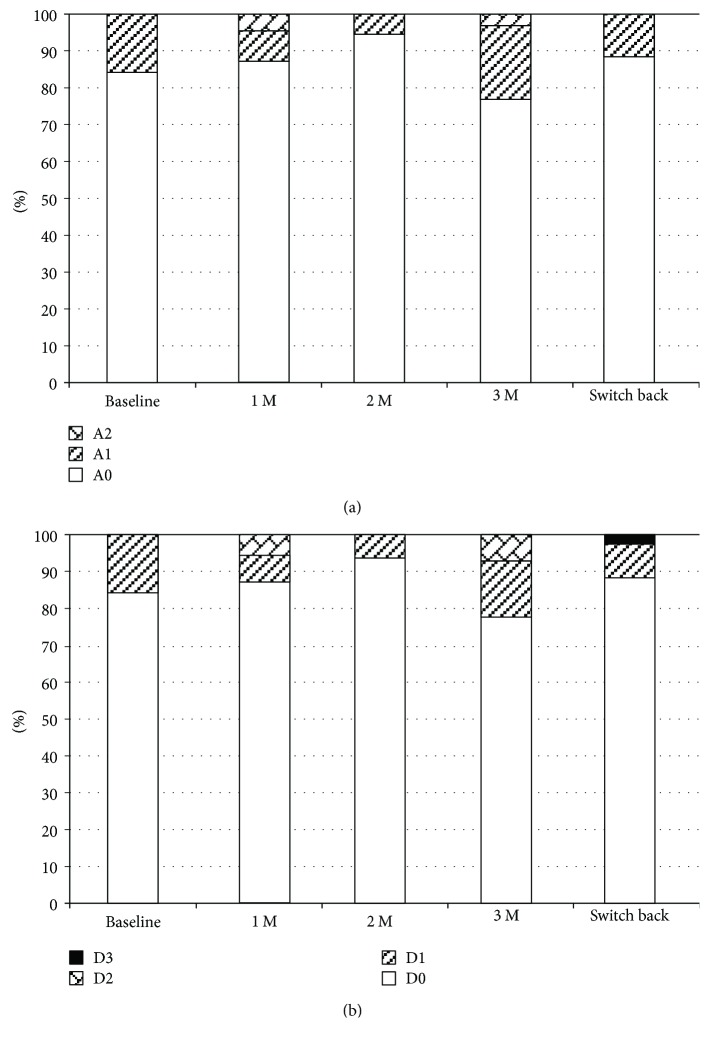
Changes in corneal epithelial defects. (a) Area of corneal epithelial defects. (b) Density of corneal epithelial defects. A: area; D: density; M: months.

Taf/Tim administration did not influence conjunctival hyperemia (Figure 5). Regarding superficial punctate keratopathy, neither area nor density significantly differed between the period before and after the switch to Taf/Tim therapy (Figures 6(a) and 6(b)). No significant differences were found in best-corrected visual acuity between baseline and 3 months after the switch to Taf/Tim (*P* = 0.35). One patient was dropped from the study due to chest pressure. Since this symptom was relieved in this patient after Taf/Tim therapy was quit, a causal relationship between Taf/Tim and chest pressure could be possible.

### 3.5. Questionnaire Survey Results

**Figure 7:**
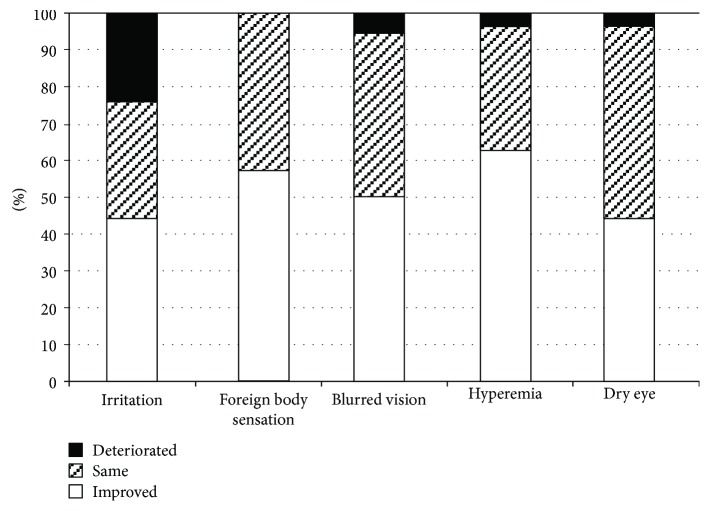
Survey one month after fixed-combination therapy introduction.

**Figure 8:**
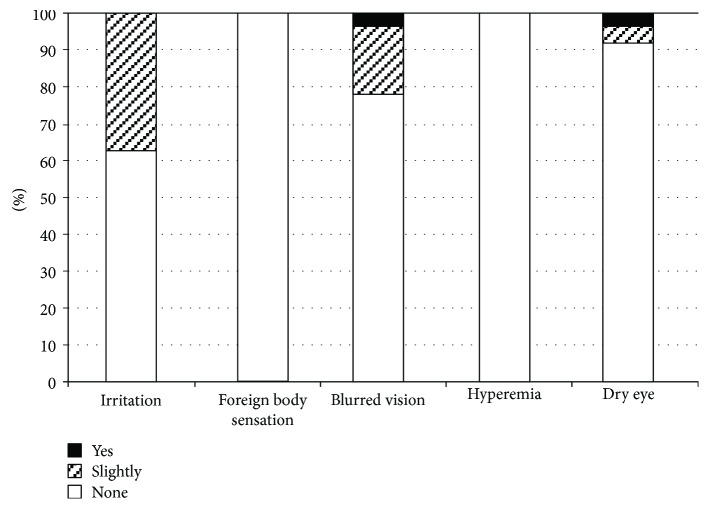
Survey three months after fixed-combination therapy introduction.

In the survey conducted 1 month after the switch to Taf/Tim from PGA monotherapy, more than 60% of patients reported improvement or no changes after the introduction to Taf/Tim therapy on all questions, and approximately half of the patients reported improvement in all symptoms after administration of Taf/Tim therapy compared to PGA monotherapy (Figure 7). Compared to the results of other questions, the result for irritation showed a significantly higher rate of deterioration (22.5%) after the introduction of Taf/Tim therapy (*P* < 0.05, contingency table analysis). In the survey conducted 3 months after the switch to Taf/Tim therapy, more than 60% of patients reported no symptoms, except for itching and blurred vision (Figure 8). Itching was the most frequent adverse effect, followed by blurred vision. Approximately 66% of patients reported no preference, 21% preferred Taf/Tim, and 13% preferred PGA.

## 4. Discussion

The clinical use of Taf/Tim was initiated worldwide in 2014, and evidence-based reports on the efficacy and safety of Taf/Tim have been limited. Okumichi et al. reported that, compared with latanoprost monotherapy, Taf/Tim significantly reduced IOP by approximately 8% [11]. The aim of our prospective, open-label, multicenter study was to assess the effect and safety of Taf/Tim combination ophthalmic solution.

If the IOP value exceeds the target level, ophthalmologists decide to change glaucoma patients' medication. However, IOP values show fluctuation due to physiological and/or pathological reasons. Therefore, the IOP value when an ophthalmologist decides to change medication may not always reflect an appropriate value. IOP values may also decrease spontaneously with no change in medication. To eliminate this possibility, patients were switched from Taf/Tim therapy back to PGA monotherapy after 3 months of treatment in this study. In our results, IOP after switching back to PGA monotherapy was small but not significantly different from that at baseline, which confirmed that our results successfully evaluated the efficacy of Taf/Tim therapy.

Ocular adverse effects sometimes result in poor adherence to treatment. Because glaucoma requires life-long treatment, treatment adherence may influence the prognosis of glaucoma. The questionnaire survey showed that symptoms, except for irritation, were improved by switching to Taf/Tim from PGAs in this study. Therefore, ensuring low rates of adverse effects is important. From this perspective, it is promising that Taf/Tim treatment did not increase the occurrence of conjunctival hyperemia or corneal epithelial damage compared to PGA monotherapy. One possible explanation for these results is the low dose of BAC used as a preservative in the Taf/Tim solution. Previous studies have reported that BAC affects the ocular surface; therefore, a BAC-free or reduced-BAC ophthalmic solution would be beneficial for corneal health.

Taf/Tim solution contains 0.001% BAC, while latanoprost solution contains 0.02% BAC. Since travoprost solution does not contain BAC, we compared the effects of Taf/Tim solution on superficial punctate keratopathy between two PGAs. Compared to both PGAs, Taf/Tim solution tended to decrease superficial punctate keratopathy, but there was no significant difference between the travoprost solution group and the latanoprost solution group. Fuwa et al. reported that Taf/Tim solution showed a lower cytotoxic effect on cultured human epithelial cells than a latanoprost/timolol fixed-combination (Lat/Tim) solution containing 0.02% BAC [12]. Okumichi et al. reported that the ocular surface safety of Taf/Tim solution was not significantly different from that of latanoprost [11].

Cho et al. [13] reported that a fixed-combination antiglaucoma ophthalmic solution was beneficial for reducing adverse ocular surface changes during long-term use. We previously compared ocular adverse effects between PGA monotherapy and travoprost-Tim fixed-combination therapy with the same protocol used in the present study, and we reported no significant difference in ocular adverse effects between the two groups [9]. In the current study, some patients complained of increased itching, blurred vision, hyperemia, and dry eye, but the majority of these symptoms disappeared by 3 months, except for itching and blurred vision. Consequently, Taf/Tim may be a better choice for long-term glaucoma treatment. Recently, a phase I study using a preservative-free Taf/Tim solution was completed [14]. However, further research is required to confirm the efficacy and safety of these new eyedrops. We conducted a survey to compare patients' preference for fixed-combination versus PG monotherapy, and the number of patients who preferred Taf/Tim was much larger than the number who preferred PGA. This result may indicate that patients accept Taf/Tim because of its better effectiveness and reduced adverse effects compared with PGAs.

Beta blocker eyedrops used in either monotherapy or fixed therapy cause a reduction in heart rate. During the 3-month period following the switch to Taf/Tim, the heart rate tended to decrease by 4.3%, but no change in blood pressure was noted. These results were similar to our previous study comparing PGA monotherapy and travoprost-Tim fixed-combination therapy [9]. Previous reports have suggested that Tim affects respiratory function in addition to heart rate [15–17]. One patient in our study cohort complained of chest pressure. Additionally, long-term use of beta blockers can lead to a subclinical increase in bronchial reactivity, even in healthy individuals [18], and reversible breathing disorders in the elderly [19]. Because all combination ophthalmic solutions currently available in Japan contain timolol or carteolol, it is necessary to carefully monitor circulatory and respiratory function when these solutions are prescribed. None of the side effects observed in our patients was serious, but careful attention should be paid to the occurrence of side effects in patients on Taf/Tim therapy.

In this study, IOP measurements were performed only during outpatient visits. We did not investigate the efficacy of Taf/Tim therapy for the nighttime reduction of IOP. Beta blockers have poor efficacy for IOP reduction at night [20], which indicates a need for further research in this area. Fuwa et al. reported that, compared with Lat/Tim solution, Taf/Tim solution increased the IOP-lowering effect duration [12]; further studies employing human patients are required to confirm this result.

In conclusion, compared with PGA monotherapy, Taf/Tim therapy significantly reduced IOP. Furthermore, patients were impressed with the results of the fixed-combination treatment. The use of fixed-combination therapy may be beneficial for optimizing glaucoma treatment by improving IOP reduction and reducing side effects.

## Figures and Tables

**Table 1:** Patient demographics.

A total number of patients	40	

Age (yrs.)	66.5 ± 10.3 (range: 48 to 86)	

Male : female	16 : 24	

POAG : NTG : OH	29 : 9 : 2	

Previous PGA (latanoprost : travoprost)	32 : 8	

Previous ocular disease	Cataract	4
	Dry eye	1
	Chorioretinal atrophy	1
	Allergic conjunctivitis	2
	Episcliritis	1

Previous ocular surgery	Trabeculectomy	2
	Phacoemulsification plus intraocular lens insertion	4

Concomitant systemic disease	Hypertension	8
	Diabetic mellitus	5
	Hyperglycemia	4

POAG: primary open-angle glaucoma; NTG: normal-tension glaucoma; OH: ocular hypertension.

## Supplementary Materials

Supplementary 1 Supplemental Figure 1: questionnaire survey 1 month after introduction of Taf/Tim solution. Taf/Tim: tafluprost/timolol.

Supplementary 2 Supplemental Figure 2: questionnaire survey 3 month after introduction of Taf/Tim solution. Taf/Tim: tafluprost/timolol.
