# Supplementary figures and images for: Efficacy and Safety of Switching Prostaglandin Analog Monotherapy to Tafluprost/Timolol Fixed-Combination Therapy
Source: J Ophthalmol. 2018 Feb 21;2018:8456764. doi: 10.1155/2018/8456764 (PMC5841029; doi:10.1155/2018/8456764)

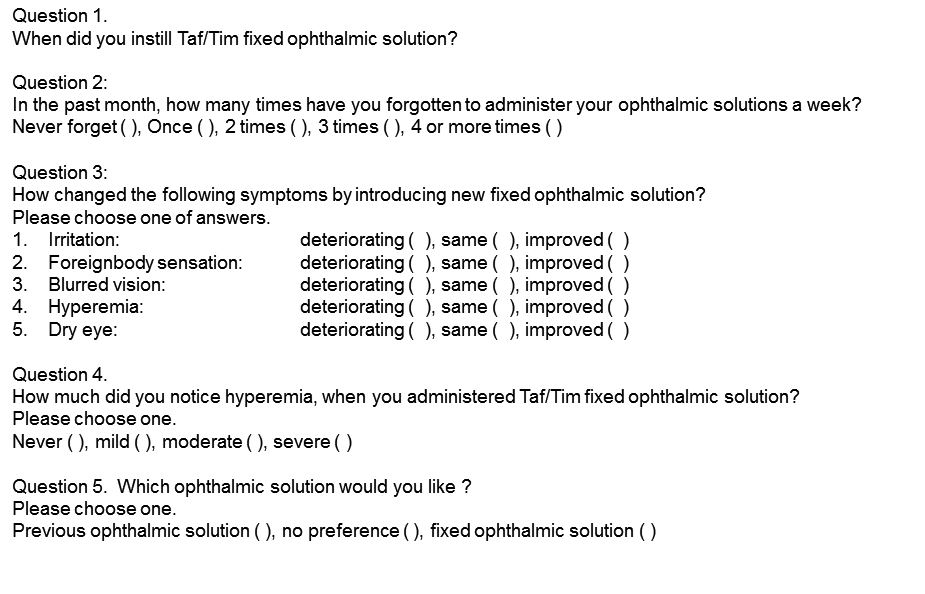

Supplement: Supplementary 1 — Supplemental Figure 1: questionnaire survey 1 month after introduction of Taf/Tim solution. Taf/Tim: tafluprost/timolol. [file 8456764.f1.tif]

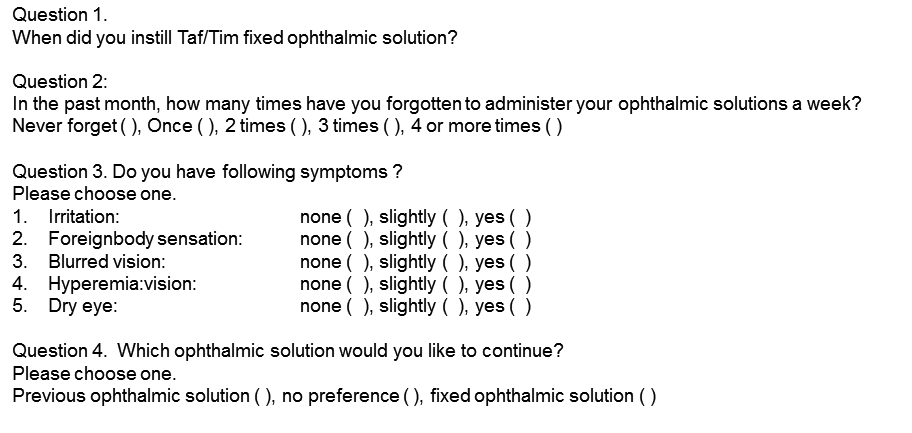

Supplement: Supplementary 2 — Supplemental Figure 2: questionnaire survey 3 month after introduction of Taf/Tim solution. Taf/Tim: tafluprost/timolol. [file 8456764.f2.tif]
